# The Influence of the COVID-19 Pandemic on the Addressability to Treatment of Children with Hyperhidrosis—A Retrospective Study and a Short Review

**DOI:** 10.3390/life14080995

**Published:** 2024-08-10

**Authors:** Florentina Nastase, Alin Codrut Nicolescu, Camelia Busila, Cristina Mihaela Marin, Elena Roxana Bogdan Goroftei, Mircea Pompiliu Bogdan, Madalina Codruta Verenca, Raisa Eloise Barbu, Alin Laurentiu Tatu

**Affiliations:** 1 Department of Neuropsychomotor Rehabilitation, ‘Sf. Ioan’ Clinical Hospital for Children, 800487 Galati, Romania; florentina34ro@yahoo.com (F.N.); madalina.verenca@ugal.ro (M.C.V.); 2 Agrippa Ionescu Emergency Clinical Hospital, 011773 Bucharest, Romania; nicolescualin66@yahoo.com; 3 Clinical Medical Department, Faculty of Medicine and Pharmacy, “Dunarea de Jos” University, 800008 Galati, Romania; cristina.marin@ugal.ro (C.M.M.); raisa.barbu@ugal.ro (R.E.B.); dralin_tatu@yahoo.com (A.L.T.); 4 “Sf. Ioan” Emergency Clinical Paediatric Hospital, 800487 Galati, Romania; 5 “Sf. Apostol Andrei” Emergency Clinical Hospital, 800578 Galati, Romania; pompiliu.bogdan@ugal.ro; 6 Multidisciplinary Integrated Center, Dermatological Interface Research MIC-DIR (Centrul Integrat Multidisciplinar de Cercetare de Interfata Dermatologica—CIM-CID), “Dunărea de Jos” University, 800201 Galati, Romania; 7 Dermatology Department, “Sfanta Cuvioasa Parascheva” Hospital of Infectious Diseases, 800179 Galati, Romania

**Keywords:** hyperhidrosis, iontophoresis, COVID-19 pandemic, quality of life

## Abstract

Introduction: This study was carried out to demonstrate the negative influence that the COVID-19 pandemic had on the ability of patients to treat hyperhidrosis with iontophoresis. The purpose of this study is to identify the annual distribution of patients with hyperhidrosis as well as elaborate a curve of cases within the time interval studied. Methods: It is a retrospective study initiated in the Department of Neuropsychomotor Rehabilitation of the “Sf. Ioan” Emergency Clinical Hospital for Children, Galati, Romania, in which we analyzed the electronic database, the treatment, and the consultation files of all the children who presented between January 2013 and December 2023. We found 111 patients who met the inclusion criteria. Results: During the 3 years of the pandemic, the number of patients who came to our clinic suddenly dropped to 0. Limitations: This study was conducted on a relatively small number of patients in a Neuropsychomotor Recovery clinic. This study includes only patients with palmar and/or plantar hyperhidrosis who presented to the clinic for iontophoresis. Conclusion: Although it is a disease that significantly influences the quality of life, patients and their families do not consider hyperhidrosis to be an urgent problem that can be improved by treatment.

## 1. Introduction

**Figure 1:**
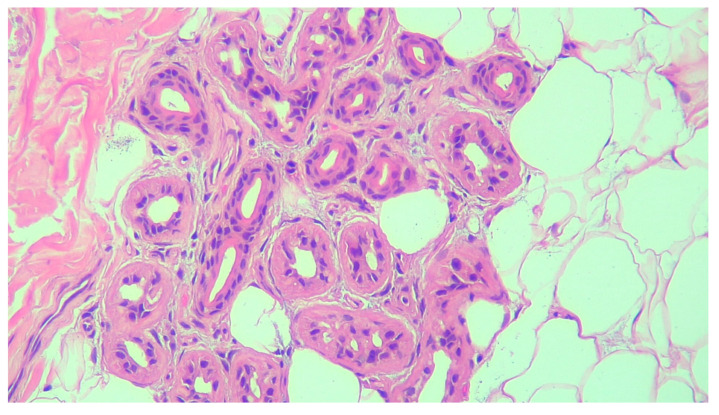
The histopathological exam of the sweat glands (picture from personal collection hematoxylin—eosin 200x).

Primary hyperhidrosis (PH) is a focal chronic autonomic skin disorder of unknown etiology with onset in childhood or puberty [1,2,3,4]. It is characterized by spontaneous excessive sweating resulting from overstimulation of the sympathetic nervous system, which disappears over sleep [1,5,6,7]. The activation of sweat glands is due to the overactivation of cholinergic receptors, and in hyperhidrosis (HH), the production of sweat is four to five times higher than normal [8]. Patients with hyperhidrosis do not show histopathological changes in sweat glands or modifications in their numbers [9]. Figure 1 shows a histopathological exam of a patient.

The temperature of the environment does not influence hyper-sweating, which is frequently accompanied by emotional and social stress [8,10]. The two types of hyperhidrosis are primary and secondary asymmetrical, generalized exaggerated sweating, which manifests during sleep and is also caused by endocrine disorders, neuroendocrine tumors, drugs, and is not associated with genetic transmission [11,12].

A complete medical history and physical examination provide all the necessary information to differentiate between the two types of hyperhidrosis. Secondary hyperhidrosis must be excluded prior. For the diagnosis of primary hyperhidrosis, the following criteria are available: at least 6 months of focal, exaggerated sweating without a cause and 2 or more of at least an episode per week; bilateral and symmetrical distribution; started before 25 years old; a positive family history; end of sweating overnight; interference in daily routine [13].

The aim of this study is to draw attention to the importance of awareness of the consequences of this disease and to provide evidence that patients and their families put its treatment on the back burner. The pandemic restrictions have a significant impact on medical or dermatological diseases, manifested by the marked reduction in face-to-face dermatological consultations and by the addressability of the treatment of patients with hyperhidrosis. This affirmation is supported in our study by the drop to 0 of patients’ presentation to treatment per year, demonstrating the impact of the pandemic on the choice to treat hyperhidrosis.

Both patients and their families, along with the medical staff they come into contact with, must realize that hyperhidrosis is a disease with a strong impact on their lives.

## 2. Materials and Methods

This is a retrospective study that was initiated after approval of the Medical Council of the “Sf. Ioan” Emergency Clinical Hospital for Children, Galati, Romania, no. 31117/04.12.2023, and it is composed of two parts. Part 1 was conducted in the Department of Neuropsychomotor Rehabilitation of the hospital between January 2014 and December 2023. Part 2 was conducted in the Dermatological Department of the hospital over the same period of time. The electronic database, the treatment, and the consultation files were analyzed. The inclusion criteria were to be diagnosed with primary hyperhidrosis and to be under 18 years old at the time of consultation. There were no exclusion criteria. After discovering the first part results, we extended the research to the Dermatological Department of the hospital and looked for the analysis of the addressability of children to the dermatologist in the same period of time.

The primary objective of this study was to identify the annual distribution of patients with primary hyperhidrosis, as well as to elaborate a curve of cases within the time interval studied and to compare with the results from the specialized literature.

## 3. Results

**Figure 2:**
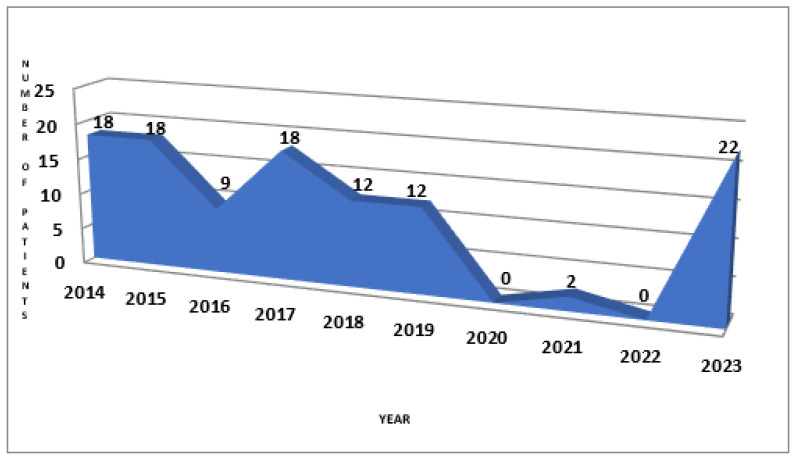
The chart of patient’s distribution per year.

Part 1: We found 111 patients who met the inclusion criteria, of which 67 (60.36%) were girls and 44 (39.64%) were boys, who were addressed to the Department of Neuropsychomotor Rehabilitation to perform iontophoresis. The ages of patients range from 6 to 17, with a mean age of 10.59 ± 2.87. We focused the research on the annual distribution of patients because we wanted to highlight the effects of the restrictions during the COVID-19 pandemic on the addressability of children to treatment. In Figure 2, it can be observed the downward slope to 0 in the 3 years in which there were restrictions and then the return to the initial number in 2023. It is important to mention that the two patients presented in 2021 had another pathology that required recovery treatment: back pain and post-humeral fracture, respectively, pathologies that required the immediate initiation of recovery. During the clinical examination of these two children, palmar sweating was also observed, and they were referred to a dermatologist to confirm the diagnosis. The distribution of the number of patients for each year studied is detailed below.

**Figure 3:**
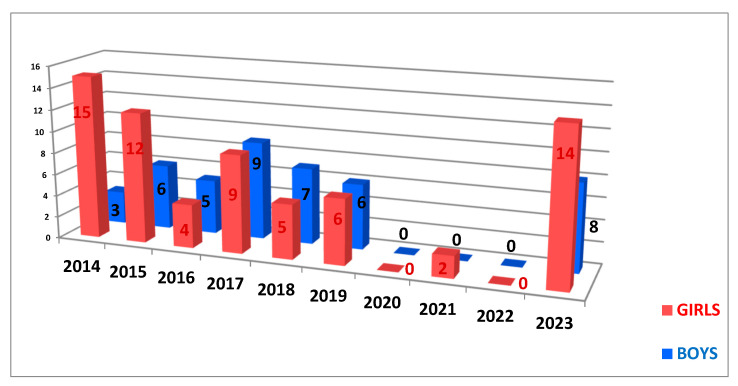
The chart of gender distribution per year.

The distribution by gender of patients differs from year to year. In the specialized literature, there is described an equality between the cases of hyperhidrosis in girls and boys, with a false predominance in girls due to their addressability and increased probability of following their treatment [13]. The same incidence is observed in our study, with a predominance of females in the first two years of the study, then an equalization of the cases, as seen in Figure 3.

The mean age of the patient group was 10.59 ± 2.87 (ranging from 6 to 17). In Table 1, the ages of the patients included in the study are detailed for each year.

Part 2: We found 180 patients who met the inclusion criteria, of which 99 (55%) were girls and 81 (45%) boys, who were addressed to the Dermatological Department to establish the diagnosis and for specialized treatment.

**Figure 4:**
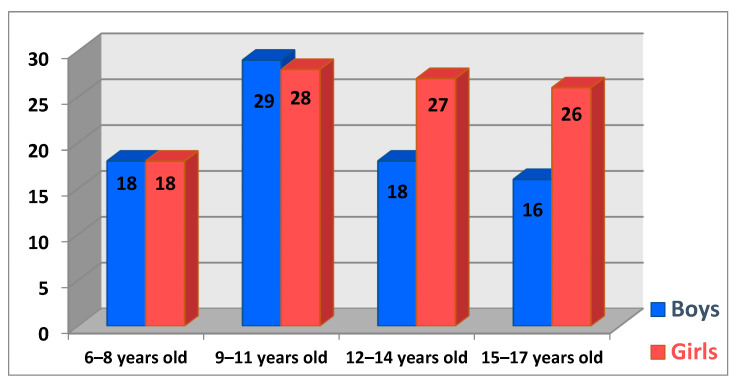
Numerical distribution of patients by age group and gender.

We chose to segment the children’s ages into four groups, starting with the 6–8-year-old segment, which allowed us to form a clear picture of the ages and identify the tendencies of each interval. The maximum incidence in this study is in the age group 9–11 years, with 31.67% of the total number of cases, which corresponds to a number of 57 children. The next age group represented by 25% (*n* = 45) of cases is the one between 12 and 14 years old, followed by 15–17-year-olds with 23.33% (*n* = 42) of patients. The last interval is represented by the 6–8-year-old patients, with 20% (*n* = 36) of patients. The distribution of patients by gender and age groups is represented in Figure 4.

We focused the research on the annual distribution of patients because we wanted to highlight the effects of the restrictions during the COVID-19 pandemic on the addressability of children to a medical consultation. After analyzing the presence of patients for each year taken into account, we observe a relatively constant trajectory in the first years, with a sudden drop to 0 in 2020—the beginning of the COVID-19 pandemic.

**Figure 5:**
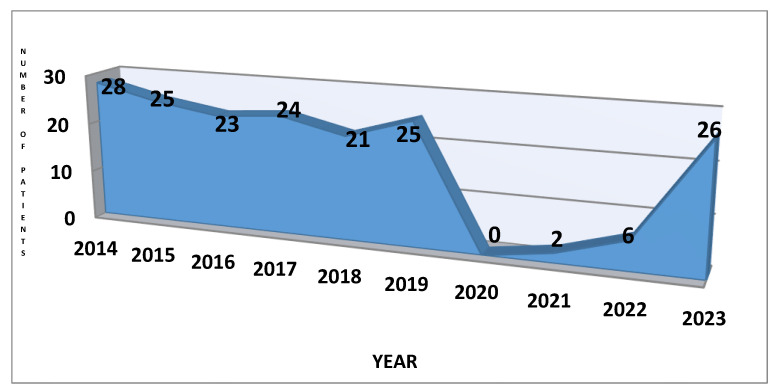
The chart of patient’s distribution per year.

In Figure 5, it can be observed the downward slope to 0 in the 3 years in which there were restrictions and then the return to the initial number in 2023. The distribution of the number of patients for each year studied is detailed below.

**Figure 6:**
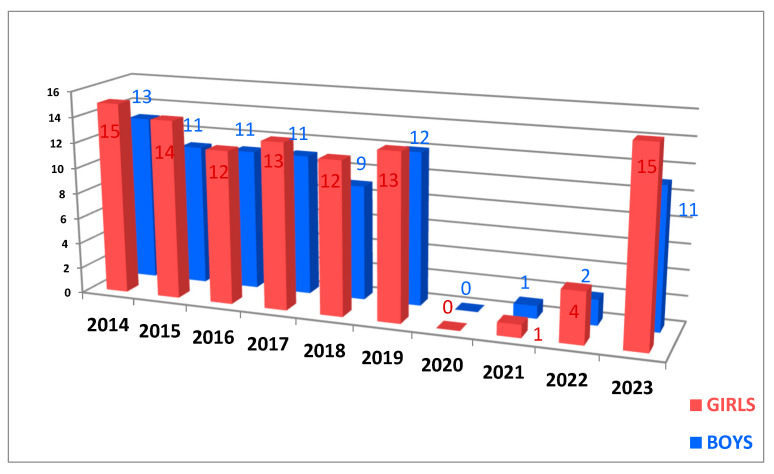
The chart of gender distribution per year.

The same observation from Part 1 about the distribution by gender of patients can also be stated in this part. In the specialized literature, there is described an equality between the cases of hyperhidrosis in girls and boys, with a false predominance in girls due to their addressability and increased probability of following their treatment [13]. The same incidence is observed in our study, with a predominance of females in the first two years of the study, then an equalization of the cases, as seen in Figure 6.

The ages of patients range from 6 to 17, with a mean age of 11.54 ± 3.06. The girls mean age is 11.84 ± 3.13, and the boys mean age is 11.17 ± 2.95. In Table 2, the ages of the patients included in the study are detailed for each year.

## 4. Discussion

The excessive sweating found in primary hyperhidrosis is spontaneous and beyond the physiological needs to maintain thermal homeostasis [5,8]. It starts during childhood and is the result of hyperactivity in the sympathetic nervous system. Although the onset is in childhood, both children and their parents are reluctant to seek medical assistance [6]. The evaluation of a patient with excessive sweating starts with a complete history and a physical examination. Laboratory testing is not necessary if the primary hyperhidrosis symptoms are characteristic; it is performed only to exclude the possible causes of secondary hyperhidrosis. The next step in the evaluation is to establish the severity of the symptoms. Gravimetric and evaporimetric tests are used in research to measure the amount of sweat. Minor’s test evaluates the size of the affected area to treat, delineating the surface, and it does not provide any information on severity [9].

The decrease in quality of life caused by hyperhidrosis is caused by embarrassment, and this disease has a severe, devastating social and emotional impact [11]. The symptoms characterized by excessive sweating have a real contribution to the deterioration of quality of life. Multiple areas of life are strongly affected, such as social, psychological, and economic areas. The symptoms can be aggravated by emotions and stress, and excessive sweating increases stress, thus forming a vicious circle [12].

In addition to quantitative tests, hyperhidrosis can also be evaluated through qualitative tests. It is imperative to measure the effects of hyperhidrosis on quality of life and any resulting impairments. For patients with primary hyperhidrosis, there are various quality-of-life questionnaires that can be disease-specific. These include the Hyperhidrosis Impact Questionnaire (HHIQ), the Hyperhidrosis Disease Severity Scale (HDSS), and dermatology-related questionnaires such as Skindex and the Dermatology Life Quality Index (DLQI). Additionally, general questionnaires like the Short Form 36 (SF-36) are also used. The most commonly used are HDSS and DLQI, frequently being used together [13].

The Hyperhidrosis Disease Severity Scale (HDSS) is a disease-specific scale that measures the severity and provides a qualitative measure based on how sweat affects daily living. It is based on one single item with four answer options, each marked from 1 to 4. The patient chooses the best-related answer regarding his experience. The score options are as follows:

- 1—indicate a mild or lack of hyperhidrosis,
- 2—indicate a moderate hyperhidrosis
- 3 and 4—indicate a severe hyperhidrosis.

Studies associate a drop of 2 points on the scale with an 80% reduction in the amount of sweating and a drop of 1 point with a 50% reduction [9,13]. Treatment success is defined as an HDSS decrease from 4/3 to 2/1 or from 2 to 1, and failure is defined as the absence of a change after one month of treatment [13].

Hyperhidrosis affects patients in many aspects of daily life. It is important to make an early diagnosis and to receive proper treatment to prevent the severe consequences on quality of life. The diagnosis is clinical, based on subjective excessive sweat, but with the available measurement tools, it is possible to identify and grade the severity and provide prompt treatment [13].

The effects of hyperhidrosis are also on skin conditions because it interferes with protective mechanisms. Hyperhidrosis patients have a greater prevalence of fungal, bacterial, or viral dermatoses [12].

COVID-19 (Coronavirus Disease 2019) is a very contagious disease caused by a virus known as SARS-CoV-2, a newly discovered one [14,15,16,17]. In March 2020, the COVID-19 pandemic was declared by the World Health Organization (WHO) until May 2023. It may cause a wide variety of persistent symptoms, not only in individuals with underlying conditions and in the elderly but also in people with no or a few chronic medical conditions and in young adults [18].

It is well known all the consequences of COVID-19. We just remember the effects on the lungs—respiratory distress syndrome and interstitial pneumonia; on the heart—heart failure; on the brain and nervous system—stroke; on mental health—depression and anxiety; and on the musculoskeletal system—fatigue. All these reasons may lead to a poorer quality of life [18] and a drastic change in life, causing mental problems such as depression, whose prevalence increased during the pandemic. Anxiety and depression in hyperhidrosis patients have been investigated, but not in a sufficient number of studies. The patients had a relatively higher degree of depression.

The prevention measures for the spread of SARS-CoV-2 had a significant impact on medical or dermatological diseases. The impact of the pandemic, through its restrictions, was manifested by the marked reduction in face-to-face dermatological consultations and by functional impairment in their quality of life [19]. Other patient complaints were related to the lack of guidance and problems with reliable information for treatment. The diseases were managed by the patients themselves, by non-medical strategies, or by self-medication. The limitation of medical supervision induced adverse events, several complications, or life-threatening problems [19,20].

The lives of billions of children and adolescents changed rapidly during the pandemic due to massive prevention measures. They face massive changes in their daily routine, such as home confinement, school closure, or social distancing. The consequences of COVID-19 had a massive impact on the quality of life and mental health of the children. Also, they received less pediatric care, resulting in diseases that remained untreated. Worries, anxiety, helplessness, and fear were the psychological distress experienced by the adolescents and children [21,22].

In the first part of the study, it was observed that the addressability of our clinic to perform iontophoresis dropped to zero in the years with COVID-19 restrictions. It turns out that patients and their families consider hyperhidrosis to be a less serious condition for which they can give up treatment. The two patients with hyperhidrosis, who were addressed to us during a pandemic in 2021, came for another pathology. They needed to start immediately the rehabilitation treatment for back pain and post-humeral fracture. During the clinical examination, we also observed that their palms were sweaty, and after the confirmation of the diagnosis by the dermatologist, they performed iontophoresis for hyperhidrosis together with the treatment for the main pathology. The pandemic affected everyone’s quality of life, especially hyperhidrosis patients whose daily activities were affected.

This study is limited by the inclusion of patients with palmar, plantar, and the palmo-plantar combination because these are the areas treated in our clinic by iontophoresis. In the future, we want to extend this study by adding a prospective one, in which we will follow the impact of hyperhidrosis on the quality of life of children.

In the second part of the study, it can be observed that the patients have the same tendency not to come to the medical service to receive treatment. In the first year of restrictions, no patient with hyperhidrosis was addressed to the dermatologist at our hospital to receive treatment.

## 5. Conclusions

1. The pandemic restrictions have a significant impact on medical or dermatological diseases.
2. The impact of the pandemic was manifested by a marked reduction in face-to-face dermatological consultations.
3. The COVID-19 pandemic also left its mark on the addressability of treatment for patients with hyperhidrosis.
4. The drop to 0 in patients’ presentation to perform iontophoresis per year demonstrates the impact of the pandemic on the choice to treat hyperhidrosis.
5. There are two patients with hyperhidrosis who came to the Rehabilitation Department in 2021; it is important to highlight that the patients and their families came for other reasons, more urgent, and they performed iontophoresis, together with the specific rehabilitation program for back pain and humeral fracture status.
6. There is also a drop to 0 in the addressability of the dermatologist for diagnosis and treatment.
7. It must be realized that hyperhidrosis is a disease with a strong impact on children’s lives.

## Figures and Tables

**Table 1:** The distribution of patients per year.

Year	*n* = 111 (%)	Girls (%)			Boys (%)		
		*n* (%)	Age		*n* (%)	Age	
			Mean ± SD	Min.–Max.		Mean ± SD	Min.–Max.
2014	18 (16.22%)	15 (83.33%)	11.20 ± 3.36	6-17	3 (16.67%)	10 ± 4.35	7–15
2015	18 (16.22%)	12 (66.67%)	10.83 ± 2.51	7–14	6 (33.33%)	10.66 ± 2.16	8–14
2016	9 (8.11%)	4 (44.44%)	10.25 ± 2.62	8–14	5 (55.56%)	10 ± 3.67	7–14
2017	18 (16.22%)	9 (50%)	11.22 ± 3.99	6–17	9 (50%)	11.33 ± 2.55	9–14
2018	12 (10.81%)	5 (41.67%)	10.6 ± 2.60	9–15	7 (58.33%)	10.71 ± 2.69	8–15
2019	12 (10.81%)	6 (50%)	9.66 ± 3.77	7–17	6 (50%)	9.83 ± 3.65	7–15
2020	0 (0%)	0 (0%)	0	0	0 (0%)	0	0
2021	2 (1.80%)	2 (100%)	11.5 ± 3.53	9–14	0 (0%)	0	0
2022	0 (%)	0 (0%)	0	0	0 (0%)	0	0
2023	22 (19.81%)	14 (63.64%)	10.28 ± 2.36	6–14	8 (36.36%)	9.62 ± 2.44	7–14

**Table 2:** The distribution of patients per year.

Year	*n* = 180 (%)	Girls (%)			Boys (%)		
		*n* (%)	Age		*n* (%)	Age	
			Mean ± SD	Min.–Max.		Mean ± SD	Min.–Max.
2014	28 (15.56%)	15 (53.57%)	11.73 ± 3.45	7–17	13 (46.43%)	11.46 ± 4.12	7–16
2015	25 (13.89%)	14 (56%)	10.78 ± 3.46	7–17	11 (44%)	10 ± 2.79	7–16
2016	23 (12.78%)	12 (52.17%)	12.75 ± 2.63	8–16	11 (47.83%)	11.18 ± 2.89	7–16
2017	24 (13.33%)	13 (54.16%)	11.15 ± 3.46	6–16	11 (45.84%)	11.63 ± 2.80	7–16
2018	21 (11.67%)	12 (57.14%)	11.75 ± 2.66	8–16	9 (42.86%)	10.33 ± 2.54	7–15
2019	25 (13.89%)	13 (52%)	13.07 ± 3.27	7–17	12 (48%)	11 ± 3.51	7–16
2020	0 (0%)	0 (0%)	0	0	0 (0%)	0	0
2021	2 (1.11%)	1 (50%)	16	16	1 (50%)	14	14
2022	6 (3.33%)	4 (66,67%)	12.25 ± 2.25	9–15	2 (33.33%)	13.5 ± 3.53	11–16
2023	26 (14.44%)	15 (57.69%)	11.46 ± 2.92	7–17	11 (42.31%)	11.72 ± 3.06	7–17

## Data Availability

The data presented in this study are available on request from the corresponding author.
